# Multi-Robot Coalitions Formation with Deadlines: Complexity Analysis and Solutions

**DOI:** 10.1371/journal.pone.0170659

**Published:** 2017-01-24

**Authors:** Jose Guerrero, Gabriel Oliver, Oscar Valero

**Affiliations:** University of the Balearic Islands, Mathematical and Computer Science Department, Palma de Mallorca, Illes Balears, Spain; Peking University, CHINA

## Abstract

Multi-robot task allocation is one of the main problems to address in order to design a multi-robot system, very especially when robots form coalitions that must carry out tasks before a deadline. A lot of factors affect the performance of these systems and among them, this paper is focused on the physical interference effect, produced when two or more robots want to access the same point simultaneously. To our best knowledge, this paper presents the first formal description of multi-robot task allocation that includes a model of interference. Thanks to this description, the complexity of the allocation problem is analyzed. Moreover, the main contribution of this paper is to provide the conditions under which the optimal solution of the aforementioned allocation problem can be obtained solving an integer linear problem. The optimal results are compared to previous allocation algorithms already proposed by the first two authors of this paper and with a new method proposed in this paper. The results obtained show how the new task allocation algorithms reach up more than an 80% of the median of the optimal solution, outperforming previous auction algorithms with a huge reduction of the execution time.

## Introduction

Systems with two or more mobile robots (multi-robot-systems) can perform tasks that with one robot would be impossible to carry out or would take much more time. Moreover, such systems are more robust, scalable and flexible than those with only one robot. However, a lot of new challenges and problems must be solved before taking advantage of the potential benefits of multi-robot systems. Among all possible issues that arise in a natural way in multi-robot systems, this paper focuses on the problem commonly referred to as “Multi-robot Task Allocation” (MRTA) which consists in selecting the best robots to execute each one of the tasks that must be performed. MRTA is still an open problem, specially when several robots (a coalition) have to execute concurrently a task before a deadline. Furthermore, in most cases, this problem is either NP or needs a very long time to provide an optimal solution. To address this problem, auction-like algorithms are widely used, where the robots negotiate between them to achieve a solution.

This paper is focused on the formation of multi-robot coalitions under an auction-based framework, where the tasks must be executed before deadlines. In this case the knowledge of how long the coalition will take to finish a task is essential in order to determine the proper alliances. The time needed to finish a task is frequently unknown because it depends on many complex and dynamic factors, among them this paper will center its attention on the physical interference between robots. The interference appears when some individuals of a community compete for a shared resource. In multi-robot systems, a frequent situation occurs when two or more robots need to access the same point simultaneously. It has been demonstrated in different studies [1, 2], that the physical interference has an important impact on the system performance. This effect can be dramatic when the system has to address tasks with deadlines because the interference increases the time needed by the coalition to finish the task and, therefore, diminishes the coalition utility.

This paper provides the first mathematical formalization of a MRTA problem with deadlines that takes into account the interference effect. Moreover, it analyzes the complexity of the resulting optimization problem by means of the coalition utility and interference. As will be demonstrated, depending on the interference function and on the task characteristics, the allocation can be faced up as an integer linear optimization problem. The interference function values have been obtained from the execution of a large number of experiments with physical simulators in the authors’ previous works [3]. Recently, other authors (see [4]) gave a mathematical model for MRTA with interference but without deadlines. Another example of the growing interest for this field is the Nunes’ et al. work [5] where a formalization of the task allocation with deadlines is given only from a theoretical point of view, without neither implementing nor testing their models. Our paper extends this work and carries out an extensive number of experiments to compare the first two authors’ previous works, given in [3], regarding to the optimum solution. Thus, the experiments show that the algorithms given in [3] can reach up to more than an 80% of the optimal solution with a very low execution time. In the light of the formalization and, as a secondary contribution of this paper, a new auction-like MRTA algorithm, called Multi Objectives Double Round Auction (MDRA), is proposed and tested. The experimental results show that this new method outperforms the previous ones.

The interference can cause a high impact when implemented in real robots and, as Rubenstein et al. points out in [6], is crucial to implement motion strategies for avoiding failures. Despite this need, this paper is only focused on MRTA problems ignoring the lower motion issues. As future work, we will address how to join both our MRTA algorithms and motion control algorithms in an unique strategy.

The rest of the paper is organized as follows: first, Section MRTA Problem Fundamentals reviews the basic formal definitions of the MRTA problem that can be found in literature; Section Previous work on coalition formation is focused on the algorithms formerly proposed to deal with multi-robot coalitions proposed in literature; Section Coalition formation complexity analysis provides a new formalization for MRTA problem that take into account interference; Section Task Allocation: Double Round Auction for tasks with deadline reviews the authors’ previous work based on auctions and, in addition, introduces the new MDRA algorithm; the experimental results are shown and analyzed in Section Experimental results and finally, conclusions and future work are presented in Section Conclusions and future work.

## MRTA Problem Fundamentals

In this section we review the fundamental aspects of multi-robot task allocation proposed in the literature. Specially, we focus on the problem of the complexity and on the relationships with other knowledge fields such as multi-agent or task-scheduling. As will be seen, most MRTA problems for the coalition formation are NP-hard, thus, making necessary the use of heuristic functions for obtaining a solution close enough to the optimum value in a moderate time. The MRTA methods can be classified in three groups: centralized approaches, swarm-based methods and auction algorithms. The last two approaches are the most used nowadays, but as will be seen, these methods present serious limitations when the robots must form coalitions to face up tasks with deadlines.

Some taxonomies for MRTA systems have been proposed in the literature, but probably one of the most broadly used is authored by Gerkey and Mataric [7]. Gerkey’s taxonomy breaks down the MRTA problem in three orthogonal axes:

- Single-Task robots (ST) vs Multi-Task robots (MT): in a ST problem every robot can only execute a task at a time. While in contrast, in a MT problem the same robot can execute several tasks simultaneously.
- Single-Robot tasks (SR) vs Multi-Robot tasks (MR): if two or more robots can collaborate to carry out the same task, the problem is MR. The group of robots assigned to a task is called coalition or working group. Otherwise, if only one robot can be assigned to each task at the same time, the problem is called SR.
- Instantaneous assignment (IA) vs. Time-Extended assignment (TE): in an IA problem, the task allocation is made without taking into account future allocations, that is, without planning the future. Conversely, in a TE problem, a planner considers the future assignments.

This taxonomy will be used to classify the different MRTA methods exposed from now on. Regardless of Gerkey’s category, the algorithms can be preemptive or non preemptive. A preemptive algorithm is a method where the robots can change their current assigned tasks before ending them.

### A single robot per task: the SR problem

This section reviews some definitions used to address the SR problem. As proved by Gerkey in [8], the ST-SR-IA algorithms can be described in terms of the well-known Optimal Assignment Problem (OAP). The OAP is defined as: given *n* agents (robots) and *m* tasks to carry out, each agent can only be assigned to one task and each task requires only one agent. For each couple (agent-task), a value is defined that forecasts the agents performance for that task, that is, this value models the agents utility regarding that task. The goal is to assign an agent to each task to maximize the total utility *U*. This goal function is given as:

$$\begin{array}{c} U=\sum_{1\leq i\leq n}\sum_{1\leq j\leq m}\alpha_{ij}U_{ij}w_{j}, \end{array}$$ (1)

where *α*_*ij*_ = 1 if the task *i* is assigned to agent *j*, and *α*_*ij*_ = 0 otherwise; *U*_*ij*_ is the utility gained by the system when agent *j* is assigned to task *i*; and *w*_*j*_ is the weight or importance of the task *j*. Thus, *w*_*j*_ represents the priority of the task. The Hungarian’s method allows to get the optimal solution for this kind of problems in a time *O*(*nm*^2^) through a dynamic programing centralized method [9]. We have to note that, if the utilities are interrelated, that is, if the agent’s utility for a task depends on the tasks previously assigned to that agent, then the assignment is a NP-hard problem of type ST-SR-TE.

### Multiple robots per task: MR problem

In this section we introduce the definitions for MR problems, where the robots can form coalitions for carrying out a task. In this case, the definition for the Set Partitioning Problem (SPP) can be given in the following way according to [10]: given a finite set *E*, a family *F* of all feasible subsets of *E* and a utility function $U:F\to R^{+}$, the goal is to find a family *X* of *F* that has the maximum utility and which is a partition of *E*. The SPP problem is NP-hard, as it was proved in [11]. The Set Covering Problem (SCP) is similar to SPP but it is enough that *X* is a covering of *E*, being this a SCP NP-complete problem [12]. Both problems can be viewed as general cases of the multi-robot coalition formation problem, where *E* is the set of robots, *F* is the set of feasible robot coalitions formed (each one with a task assigned), and *U* is the utility function assigned to each coalition [13]. If time constraints are needed, the task deadlines should be considered just as one of the conditions to validate a family *F* of subsets of *E*. On the one hand, the SCP definition allows to allocate more than one task to the same robot and, therefore, it can address MT problems. On the other hand, the SPP definition is more restrictive because each robot can only belong to a single coalition at the same time and, therefore, these problems are ST. In [14], it is proved that, using the SPP and SCP definition, the multi-robot coalition formation problem is not only NP-hard but, in general, it cannot be approximated by a factor *O*(*m*^1−*ε*^), for any *ε* > 0, where *m* is the number of tasks. This means that, unless P = NP, the solution obtained by means of an algorithm with a complexity bounded by a polynomial function will always be *O*(*m*^1−*ε*^), for any *ε* > 0 worst than the optimal solution. As can be observed, the complexity of the coalition formation algorithms is very high. This fact justifies the great number of *ad—hoc* solutions that can be found in the literature.

In [15], a new coalition formation algorithm is proposed, based on the SCP and SPP definitions, where the maximum number of agents per coalition (*k*) is fixed beforehand. The algorithm complexity is *O*(*n*^*k*^*m*), where *n* is the number of agents and *m* the number of tasks. In [13], it is described a new algorithm with a complexity *O*(*n*^*k*^*m*) that extents to multi-robot systems the previous algorithms proposed only for multi-agents in [15]. In [14], Service and Adams improve the above cited algorithm given in [13], with a $O(n^{\frac{3}{2}}m)$ complexity. In any case, this solution is, in general, not optimal yet. Only, if the robots can be grouped in *j* homogeneous groups, in such a way that each robot only belongs to one of these groups, Service and Adams’ algorithm provides an optimal solution, with a complexity equal to *O*(*n*^2*j*^*m*). In this algorithm, the task must be assigned to a certain number of robots to start its execution and only when this number is reached the task *i* is executed with a utility *u*_*i*_. In nex sections, a new method, based on the SPP’s definition, will be explained where the number of robots *k* do not need to be known beforehand, the robots are not homogeneous and the utility *u*_*i*_ depends, among other factors, on the coalition size. Therefore, Service and Adams’ algorithm is not suitable for these kind of environments. It is also remarkable the Agarwal et al. contribution [16], where genetic algorithms optimize multiple objectives simultaneously for the coalition formation. These objectives can be the distance traveled by the robots, the number of finished tasks, or any other system parameter. Due to its complexity, the evolutive algorithms cannot deal with neither dynamic tasks nor tasks with time restrictions. Nevertheless, the Agarwal’s evolutive methods are very good candidates to improve someway the real time system’s performance.

### Scheduling: ST-MR-TE Problems

The above definitions, based on SPP or SCP, do not take into account any temporal condition for the tasks, that is, they cannot deal with any scheduling consideration. On account of [17], the coalition formation problem can be defined in terms of the Job Shop Scheduling (JSS) problem as follows: given a set of *m* jobs *J* = {*J*_1_, *J*_2_, …, *J*_*m*_} and a set of *n* machines, each job *J*_*i*_ has *m*_*i*_ associated operations *O*_*i*_ = {*O*_*i*1_, *O*_*i*2_, …, *O*_*im*_*i*__} each one of them with an execution time for each machine *T*_*ij*_. The system’s goal is to decide (schedule) in which order the tasks must be carried out on the machines to minimize the execution time. Moreover, this formulation allows defining a precedence order among operations and, even more, set a maximum execution time bound for the tasks. Balas et al. in [18] propose a method that take into account all these constraints in a multi-agent scenario. It was demonstrated that, in general, the JSS problem is NP-hard. The JSS problem can be adapted to a multi-robot problem in such a way that the system should decide the execution order of the tasks assigned to each robot to minimize, among other factors, the total execution time, the traveled path or the robots’ energy consumption. Thus, these problems are classified as TE, following the Gerkey’s taxonomy. For example, Jones et al. in [19] use genetic algorithms to solve a variation of the JSS problem for multi-robot systems.

The Vehicle Routing Problem with Time Windows (VRPTW) can also be considered as a scheduling problem, where a set of vehicles must transport goods to several “clients”. Each vehicle has a load capacity and each client demands a quantity of goods that have to be delivered within a time window. The system goal is to find a route, as short as possible, that satisfies the clients constraints with the fewest number of vehicles [20]. As the JSS definition, the VRPTW problem is NP-hard too. A variation of the VRPTW is the Tasks with Overlapping Time Window Problem (TOTWP) where the time window can be overlapped. Nunes et al. in [21] try to solve the TOTWP but with non realistic environments and with a single robot per task (SR problem).

Finally, it should be noticed that MT problems, where a robot is able to execute several tasks at the same time, are not feasible in most of the multi-robot missions because, in general, the robots must be located in an specific place to carry out a task.

## Previous work on coalition formation

This section focuses on the coalition formation methods that can deal with task deadlines. Depending on how the task allocation process is distributed among the robots, the coalition formation methods can be classified into the following categories: centralized systems, self-organized approaches (swarm intelligence) and auction methods. The Gerkey’s taxonomy will be applied into each one of these groups.

### Centralized systems

In the centralised systems all the information about the robots or the environment characteristics is sent to a central agent that takes all the decisions. Typically, these systems use techniques such as, linear programming or heuristic searches on graphs to find the optimal solution or the closest to the optimal one. Koes et al. [22] utilize linear programming to address simultaneously task allocation, path planning and task scheduling. Yu and Cai, in [23], propose a centralized mechanism called Heterogeneous Interactive Cultural Hybrid Algorithm (HICHA) where, among other techniques, use genetic algorithms. Another centralized system has been introduced by Smith and Bullo in [24], where different task allocation methods are analyzed as a function of the arrival frequency of the tasks.

### Swarm Intelligence

Swarm methods are inspired by insect colonies behavior, such as bees or ants, where a global action emerges from the interaction between very simple entities. These methods belong to a more general category of systems called self-organized systems which are defined by Cao et al. [25] as follows: *“[…] systems of non-intelligent robots exhibiting collectively intelligent behavior”*. That is, they consist on system without any central node, where the cooperative behavior emerges from the interaction of very simple behaviors running on each robot.

The main problem of swarm systems is its lack of global knowledge about the decision made by other robots. Due to this fact, the solutions provided by the swarm methods are far away from the optimal one, moreover, they can only address SR problems of limited complexity such as foraging or cleaning with homogeneous robots. This restriction is superseded in some already explained swarm systems by the authors [26, 27]. Due to its communication complexity, Low’s et al. algorithm [27] does not show some advantages of swarm methods. Furthermore, in Low’s system the robots must be homogeneous, and it cannot handle task time restrictions.

One important issue is how swarm system manage the physical interference effect between robots. This aspect, that will be addressed in Section Physical interference between robots, has already been broadly faced up in several papers, such as [28, 29]. Khaluf’s swarm methods [30] can manage deadlines but without taking into account the interference effect.

### Auction methods

Auction methods use communication protocols between robots to implement an explicit negotiation about which task must execute each robot or coalition of robots. These protocols are based on the Contract Net Protocol (CNP) proposed by Smith in 1980 [31]. Smith’s work defines a mechanism to assign tasks to communication nodes (machines) connected between them, that must solve some cooperative problem in a distributed way. When a node, called manager or auctioneer, finds or generates a new task, sends a message to announce it before starting the execution. The other nodes, called bidders, send to the auctioneer a value, called bid, that indicates how suitable are they for executing the new task. Once the manager node has received all the bids, it selects the best node (node with the highest bid) for the task. In multi-robot systems, the nodes are the robots and the manager is a central agent or another robot.

Most current auction methods only admit a robot per task, therefore, they are SR methods. In contrast, MR problems, which require coalition formation mechanisms, have hardly been addressed by auction approaches. Vig and Adam’s RACHNA method [32] allows MR problems but without deadlines. Paquet in [33] proposes a method where the working group size is unknown beforehand and considers task deadlines. This mechanism has only been tested on multi-agent systems, such as Robocup competition, but not on real or simulated physical robots. Furthermore, due to the high computational cost of its algorithms, the decision about how many agents are needed to finish a task cannot be made on-line. In [34], Tang and Parker introduce another auction approach, called ASyMTRe, that allows high tight tasks and coalition formation. More auction methods that admit coalition formation can be found in [35]. None of these methods consider the physical interference or the task deadlines.

Among the auction methods that allow time restrictions, one of the most remarkable work is that proposed by Jones [36]. This approach makes use of a learning algorithm to find the most suitable value for the bids in MR problems. Jones’ method uses a learning Support Vector Regression (SVR) model to obtain the bid value, but does not estimate the execution time. Lemaire’s auction method [37] allows soft deadlines but with a single robot per task. In [38], the proposed method can solve the VRPTW without overlapping windows. In [39], Campbell introduces a method that, that is partially inspired by auctions, where the robots can not decide to bid for a task despite it was the best option at that moment. The Campbell’s experimental results show a great improvement regarding the aforementioned methods.

Heretofore, all the methods cited does not try to forecast the expected execution time of the task. Sellner’s and Simmons’ SR method [40] predicts this time making use of kernels, but no physical interference is taken into account. In contrast, Sellner’s approach requires very heavy computational algorithms to carry out the tasks. As has been mentioned in previous sections, Nunes et al. [21] allow overlapped time windows with auctions to address SR problems. Finally, other auction-like mechanisms [41] also face up tasks with deadlines but with only one robot per task.

As has been exposed, the auction mechanisms allow the distribution of the decision process among the robots through communication protocols. Thus, the typical problems produced in centralized systems, such as single point of failure or overloading of the central agent, are avoided. Furthermore, these algorithms are, in general, much more simple than the centralized approaches. In contrast, to the aforesaid advantages, the solutions provided by the auction methods require more time and increase the energy consumption compared to the centralized systems. In addition, the auction methods are more complex than the swarm ones because they require communication protocols between robots. Despite of that, the auction systems outperform the swarm-based ones increasing the number of finished tasks and reducing the energy consumption, among other factors. Furthermore, due to the inter-robot communication protocols, the auction methods can deal with much more complex and tight tasks.

### Physical interference between robots

The physical interference between robots is one of the most fundamental problems to address by the task allocation mechanisms. In [2] Rosenfeld et al. clearly state the huge impact of the physical interference on the multi-robot systems performance. There are a lot of methods that try to minimize the interference effect in multi-robot systems without deadline (see, for instance, [42]).

In [43], Dahl et al. propose a mechanism called Vacancy Chains to carry out foraging tasks for MR problems. The system assigns robots to a task (transport an object that can be divided in small parts) until it detects that the amount of transported object weight decreases. This reduction can only be due to the interference between robots. Dahl’s method neither deals with tasks with deadlines nor can estimate tasks execution time. Finally, Lerman’s et al. studies [1] and Nam and Shell [44] present methods to model the interference but none of them deal with time restrictions.

Other authors describe swarm-like strategies to deal with the physical interference in systems with a huge number of robots (see [6, 45, 46]). For example, Rubensteinin et al. in [45] use self-assembly with thousand of robots to build complex structures.

## Coalition formation complexity analysis

The MR problem with deadlines has not been properly addressed by previous task allocation approaches. Moreover, no formal analysis regarding the complexity of this problem has been proposed. This section presents a formal definition of the MRTA-ST-MR with deadlines problem from which we will propose a classification, according to the computational complexity under different situations.

### Generic problem

The MRTA-ST-MR with deadlines problem (from now on simply MRTA problem) can be seen as a particular case of the SPP. From now on, N will denote the set of positive integer numbers. Moreover, in the following, *R* will denote the set of robots with *R* = {*r*_1_, …, *r*_*N*_*r*__} and *T* will denote the set of tasks to carry out with *T* = {*t*_1_, …, *t*_*N*_*t*__}, where $N_{r},N_{t}\in N$. Furthermore, we will assume that both sets, *R* and *T*, are kept constant over the time. Next, denote by *G* the the subset of 2^*R*^ formed by all working groups or coalitions of robots allowed over *R*, where 2^*R*^ stands for the power set of *R*. The criteria to decide if a working group is allowed depends on specific task characteristics.

Now, taking into account the preceding notation, we introduce the main notions in order to formalize the MRTA problem with deadlines. To this end, let us denote by $R^{+}$ the non negative real numbers.

**Definition 1** *Let t*_*j*_ ∈ *T and* $n(t_{j})\in N$ *with* 1 ≤ *j* ≤ *N*_*t*_. *The sets of coalitions assigned to t*_*j*_ *over the time*, *Sg*_*t*_*j*__, *is defined by*

$$\begin{array}{c} Sg_{t_{j}}=\left\{\left(g_{1},\tau_{1}\right),\ldots ,\left(g_{n}\left(t_{j}\right),\tau_{n}\left(t_{j}\right)\right)\right\}, \end{array}$$

*where each pair* $\left(g_{k},\tau_{k}\right)\in {\left(G\times R^{+}\right)}^{2}$ *stands for the coalition of robots g*_*k*_ *allocated at task t*_*j*_ *at time τ*_*k*_ *for all k* = 1, …, *n*(*t*_*j*_) *and*, *in addition*, *satisfies that τ*_*k*_ < *τ*_*m*_ *whenever k* < *m* *for all k*, *m* = 1…*n*(*t*_*j*_).

It must be pointed out that each coalition *g*_*k*_ allocated at a task *t*_*j*_ is kept constant from time instant *τ*_*k*_ until *τ*_*k*+1_ for all *k* = 1, …, *n*(*t*_*j*_), where *τ*_*n*(*t*_*j*_)+1_ denotes the finishing time for that task provided that the set of coalitions *Sg*_*t*_*j*__ is able to carry out the task.

Notice that if the allocation is non preemptive, that is, a robot cannot leave its current working group after allocating it in a task, then the cardinality of the set of coalitions must be lower or equal to 1, i.e., ∣*Sg*_*t*_*j*__∣ ≤ 1 for all *j* = 1…*N*_*t*_ and, thus, $Sg_{t_{j}}\in G\times R^{+}$ for all *j* = 1, …, *N*_*t*_.

**Definition 2** *Let j* ∈ {1, …, *N*_*t*_}. *Then the execution time of the task t*_*j*_ ∈ *T is defined as the function* $\beta_{j}:Sg_{t_{j}}\to R^{+}\cup \left\{\infty \right\}$ *such that*

$$\begin{array}{c} \beta_{j}(Sg_{t_{j}}))=\left\{\begin{array}{cc} \tau_{n(t_{j})+1} & \text{if}\;Sg_{t_{j}}\text{is}\;\text{able}\;\text{to}\;\text{carry}\;\text{out}\;\text{the}\;\text{task}\;t_{j}\\ \infty & otherwise \end{array}\right.. \end{array}$$

Note that *β*_*j*_(*Sg*_*t*_*j*__)) provides the time needed by the set of coalitions to finish the task *t*_*j*_ whenever the aforementioned set is able to carry out *t*_*j*_.

As has been pointed out in Section Previous work on coalition formation, giving the exact value of *β* function for a certain coalition is often impossible or quite difficult because of, among other factors, the interference effect. Therefore, in most cases, the function *β* must be predicted through different methods.

**Definition 3** *Let j* ∈ {1, …, *N*_*t*_}. *Then the utility of a task t*_*j*_ *is defined as a function* $U_{j}:R^{+}\cup \left\{\infty \right\}\to R^{+}$ *such that U*_*j*_(∞) = 0.

**Fig 1:**
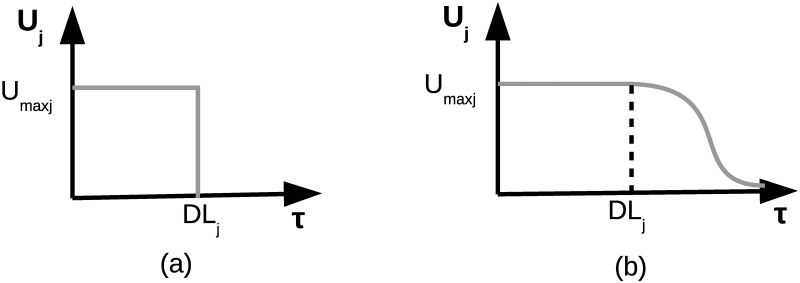
Utility function (*U*_*j*_) examples. (a) Hard-deadline utility function, (b) Soft-deadline utility function, where *τ* stands for the time needed by the coalition to finish the task.

Note that the utility function of a task will depend on task’s characteristics such as its deadline which will be denoted by *DL*_*j*_. Fig 1(a) shows an example of an utility function *U*_*j*_ called hard-deadline utility function, where *U*_*j*_(*τ*) is constant and equal to *U*_*max*_*j*__ if *τ* < *DL*_*j*_ and abruptly drops to zero if *τ* ≥ *DL*_*j*_ (the task cannot be executed on time). Another kind of utility function are so-called soft-deadline utility functions. An example of these type of functions can be seen in Fig 1(b), where the value of *U*_*j*_(*τ*) progressively falls to zero from *τ* = *DL*_*j*_.

In the light of Definition 3 it is clear that the value *U*_*j*_(*β*_*j*_(*Sg*_*t*_*j*__)) yields the utility after assigning a coalition set *Sg*_*j*_ to a task *t*_*j*_ ∈ *T*. Depending on the characteristics of the composition function *U*_*j*_ ∘ *β*_*j*_, as for instance linearity or continuity, the allocation problem must be addressed by a different kind of strategy, as will be seen later.

As we have noticed before, $Sg_{t_{j}}\in G\times R^{+}$ whenever the allocation is not preemptive. Hence in this case the global utility associated to the MRTA problem matches up with the function $U:{\left(G\times R^{+}\right)}^{N_{t}}\to R^{+}$ given as follows:

$$\begin{array}{c} U\left(Sg_{t_{1}},\ldots ,Sg_{N_{t}}\right)=\sum_{j=1}^{N_{t}}U_{j}\left(\beta_{j}\left(Sg_{t_{j}}\right)\right). \end{array}$$ (2)

It is clear that an element $\left(Sg_{t_{1}},\ldots ,Sg_{N_{t}}\right)\in {\left(G\times R^{+}\right)}^{N_{t}}$ can be identified with a function $TA:T\to {\left(G\times R^{+}\right)}^{N_{t}}$ such that

$$\begin{array}{c} TA\left(T\right)=(TA_{1}\left(t_{1}\right),\ldots ,TA_{N_{t}}\left(t_{N_{t}}\right)), \end{array}$$

where *TA*_*j*_(*t*_*j*_) = *Sg*_*t*_*j*__ for all *j* = 1, …*N*_*t*_.

Under the preceding assumptions, the goal of the MRTA problem is to maximize the global utility function, that is, to find a function TA* which maximizes the function *U*. Thus, the aforesaid goal matches up with obtaining the solution to the following optimization problem:

$$\begin{array}{c} \begin{array}{cc} & \underset{TA}{\text{maximize }}\sum_{j=1}^{Nt}U_{j}\left(\beta_{j}\left(TA_{j}\left(T\right)\right)\right)\\ & \text{subject}\;\text{to}\\ & TA_{i}\left(t_{i}\right)\cap TA_{j}\left(t_{j}\right)=\emptyset \;\text{for}\;i,j=1,\ldots ,N_{t} \end{array} \end{array}$$ (3)

Observe that we need to consider the restriction of Problem 3, since the MRTA problem is an SPP problem.

To validate the model explained above we propose a simple but generic example of a foraging-like task. To this end, we assume, in addition to the non preemptive allocation assumption, an IA strategy and that the coalitions for the tasks are formed simultaneously, i.e., *τ*_1_ is the same for all *t*_*j*_ with *j* =, …, *N*_*t*_.

#### Example: Foraging with tasks’ deadline

Foraging is one of the canonical tasks utilized by a lot of authors to state their algorithms. Moreover, it can easily be extended to other kind of missions, such as area coverage or repeated coverage.

In a foraging task there is a set of objects or tasks (*T* = {*t*_1_, …, *t*_*N*_*t*__}) to process. Each task *t*_*j*_ ∈ *T* has a workload (*L*_*j*_) that, for example, is the weight of the object; and a utility function (*U*_*j*_) with a deadline (*DL*_*j*_) (see the paragraph about utility functions with deadlines below Definition 3). Each robot *r*_*i*_ ∈ *R* = {*r*_1_, …, *r*_*N*_*r*__} has a work capacity, *C*_*ij*_, associated to each task *t*_*j*_ that represents the amount of work (object’s workload) that the robot can process per time unit. For example, in a foraging task, *C*_*ij*_ would be the object’s weight that a robot can carry per time unit. Each coalition of robots *g* ∈ *G*, in charge of a task *t*_*j*_, has a work capacity *GC*_*gj*_, that gives the amount of work that the group of robots can perform per time unit. Thus, the expected time to finish a task performed by a group of robots *g* (function *β*_*j*_) is denoted by:

$$\begin{array}{c} \beta_{j}\left(g\right)=\frac{L_{j}}{GC_{gj}} \end{array}$$ (4)

The *L*_*j*_ is known before executing the task, for example, the weight of an object to be transported or the area of a region to be covered by the robots’ group. However, it is hard to determine precisely the *GC*_*gj*_ because it usually depends on dynamic and a priori unknown factors. It is widely accepted that the factor with the strongest effect is the physical interference, as pointed out in Section Physical interference between robots. In this work the deviation from the ideal capacity of a robot’s team is summarized in a single term called the interference factor defined by a function I:G×T→R (R stands for the real numbers set), where *I*(*g*, *t*_*j*_) = *I*_*gj*_ denotes the interference effect of executing task *t*_*j*_ by a coalition of robots *g*. *I*_*gj*_ must be viewed as a noise of the ideal model (*idealCapacity*_*gj*_) that is able to include the physical interference or any other kind of perturbations, such as odometry errors (see [3].) Thus, the work capacity of a group to perform a task *t*_*j*_ is considered to be:

$$\begin{array}{c} GC_{gj}=idealCapacity_{gj}-I_{gj}, \end{array}$$ (5)

where *idealCapacity*_*gj*_ is the ideal group capacity. When the individual characteristics of the robots are known a priori, the *idealCapacity*_*g*,*j*_ can be easily estimated as follows:

$$\begin{array}{c} idealCapacity_{gj}=\sum_{1\leq i\leq n_{g}}C_{ij}, \end{array}$$ (6)

where *n*_*g*_ is the number of robots of the group *g*. This is a simple but very effective model, as has been proved by the first two authors in a previous work in [3].

From all these definitions the objective function to maximize can be defined in two different ways as follows:

If the there is no interference, that is *I*_*gj*_ = 0 for all *g* ∈ *G* and for all *j* = 1, …, *N*_*t*_, then the utility function, provided by Eq (2), is given now by

$$\begin{array}{c} \sum_{j=1}^{N_{t}}U_{j}\left(\frac{L_{j}}{\sum_{i=1}^{N_{r}}\alpha_{ij}C_{ij}}\right). \end{array}$$ (7)

Hence the Problem (3) becomes the following optimization problem:

$$\begin{array}{c} \begin{array}{cc} & \underset{\alpha_{ij}}{\text{maximize }}\sum_{j=1}^{N_{t}}U_{j}\left(\frac{L_{j}}{\sum_{i=1}^{N_{r}}\alpha_{ij}C_{ij}}\right)\\ & \text{subject}\;\text{to}\\ & \sum_{j=1}^{N_{t}}\alpha_{ij}\leq 1\;\text{for}\;i=1,\ldots ,N_{r} \end{array}, \end{array}$$ (8)

where

$$\begin{array}{c} \alpha_{ij}=\left\{\begin{array}{cc} 1 & \;\text{if}\;\text{robot}\;r_{i}\;\text{is}\;\text{assigned}\;\text{to}\;\text{task}\;t_{j}\\ 0 & \;\text{otherwise} \end{array}\right.. \end{array}$$ (9)

Notice that the restriction in Problem (3), *TA*_*i*_(*t*_*i*_)∩*TA*_*j*_(*t*_*j*_) = ∅ for all *i*, *j* = 1, …, *N*_*t*_, have been rewritten in terms of *α*_*ij*_ in order to develop our numerical schemes later on.

If there is interference then the utility function, provided by Eq (2), is given now by

$$\begin{array}{c} \sum_{j=1}^{N_{t}}U_{j}\left(\frac{L_{j}}{\sum_{i=1}^{N_{r}}\alpha_{ij}C_{ij}+I\left(TA_{j}\left(t_{j}\right),t_{j}\right)}\right). \end{array}$$ (10)

So the Problem (3) becomes the following optimization problem:

$$\begin{array}{c} \begin{array}{cc} & \underset{\alpha_{ij}}{\text{maximize }}\sum_{j=1}^{N_{t}}U_{j}\left(\frac{L_{j}}{\sum_{i=1}^{N_{r}}\alpha_{ij}C_{ij}+I\left(TA_{j}\left(t_{j}\right),t_{j}\right)}\right)\\ & \text{subject}\;\text{to}\\ & \sum_{j=1}^{N_{t}}\alpha_{ij}\leq 1\;\text{for}\;i=1,\ldots ,N_{r} \end{array}. \end{array}$$ (11)

The method under consideration to face up the Problems (8) and (11) depends on the characteristics of the composed function (*U*_*j*_ ∘ *β*_*j*_). Next we summarize, on the one hand, the optimization methods that we will use to solve the aforesaid problems and, on the other hand, the range of considereded characteristics of the utilities functions.

### Case studies

#### Case 1 (Linear and soft-deadline utility function)

*If U*_*j*_ *is a soft-deadline linear*, *continuous and decreasing function for all j* = 1, …, *N*_*t*_, *then*, *in general*, *Problems* (8) *and* (11) *becomes binary integer nonlinear optimization problems*. *This is due to the fact that the functions β*_*j*_, *given by* Eq (4), *are not linear*. *For example*, *if the utilities functions are defined by U*_*j*_(*τ*) = −*τ* + *U*_*max*_*j*__ *for all* $\tau \in R^{+}$ (*Umax*_*j*_ *is a constant for each j* = 1, …, *N*_*t*_), *then* Problem (11) *can be expressed as follows*:

$$\begin{array}{c} \begin{array}{cc} & \underset{\alpha_{ij}}{\text{maximize}}\sum_{j=1}^{N_{t}}\left(-\frac{L_{j}}{\sum_{i=1}^{N_{r}}\alpha_{ij}C_{ij}+I\left(TA_{j}\left(t_{j}\right),t_{j}\right)}+U_{max_{j}}\right)\\ & \text{subject}\;\text{to}\\ & \sum_{j=1}^{N_{t}}\alpha_{ij}\leq 1\;\text{for}\;\text{all}\;i=1,\ldots ,N_{r} \end{array} \end{array}$$ (12)

#### Case 2 (Soft-deadline utility function and *U*_*j*_ ∘ *β*_*j*_ linear)

*If the functions U*_*j*_ *are given*, *for all j* = 1, …, *N*_*t*_, *as follows*

$$\begin{array}{c} U_{j}\left(\tau \right)=\frac{Umax_{j}}{\tau } \end{array}$$ (13)

(*again Umax*_*j*_ *is a constant for each j* = 1, …, *N*_*t*_) *and I*_*g*_ *is linear*, *then it is not hard to see that* Problem (11) *is an integer linear optimization problem*, *as will be demonstrated in following paragraphs*. *This utility function form fits a great number of realistic tasks such as foraging toxic wastes where as the time passes*, *the damage of the environment increases and therefore the utility of the function also decreases*.

*In general the interference function is not linear with respect to the number of robots*, *as proved in* [2]. *However*, *in some cases this assumption is pretty realistic*, *as the experimental results of Section Experimental results demonstrate*. *Of course whenever the interference function is linear it has the following form*:

$$\begin{array}{c} \begin{array}{c} I\left(g,t_{j}\right)=a_{j}n_{g}+b_{j}, \end{array} \end{array}$$ (14)

*where we recall that n*_*g*_ *is the number of robots of the coalition g and*, *in addition*, *a*_*j*_, *b*_*j*_ *are constants that depend on the tasks characteristics*. *Section Experiments with physical robots: Interference function shows how a*_*j*_ *and b*_*j*_ *can be obtained*. *Taking into account the preceding information*, Problem (11) *can be rewritten as follows*:

$$\begin{array}{c} \begin{array}{cc} & \underset{\alpha_{ij}}{maximize\;}\sum_{j=1}^{N_{t}}\sum_{i=1}^{N_{r}}\left(U_{max_{j}}\alpha_{ij}\left(\frac{C_{ij}-a_{j}}{L_{j}}\right)\right)-Umax_{j}\frac{b_{j}}{L_{j}}\\ & subject\;to\\ & \sum_{j=1}^{N_{t}}\alpha_{ij}\leq 1\;\text{for}\;\text{all}\;i=1,\ldots ,N_{r} \end{array}. \end{array}$$ (15)

*Therefore*, *we conclude that in this case the optimization problem can be addressed*, *under the considered assumptions*, *as an integer linear programming problem*.

#### Case 3 (Hard-deadline utility function)

*If the utility functions* *U*_*j*_, *for all* *j* = 1, …, *N*_*t*_, *are two-step hard deadline discontinuous function (see* Fig 1(a)*) then we show that the problem can be addressed as an integer linear programming problem*. *To this end*, *let us recall that according to* [47] *a renowned classical optimization problem consists in minimizing a function* *f* *defined by*

$$\begin{array}{c} f=\sum_{j=1}^{N_{t}}H\left(\frac{L_{j}}{\sum_{i=1}^{N_{r}}\alpha_{ij}\left(C_{ij}-a_{j}\right)-b_{j}}-DL_{j}\right), \end{array}$$ (16)

*where each* *α*_*ij*_ *is given as in* Eq (9) *and* *H* *is the heaviside function defined by*

$$\begin{array}{c} \;H\left(x\right)=\left\{\begin{array}{ccc} 0 & \text{if} & x<0\\ 1 & \text{if} & x\geq 0 \end{array}\right.. \end{array}$$ (17)

*Following* [47], *the preceding optimization problem can be converted into an integer program problem adding for each task* *t*_*j*_ *a new binary auxiliary variables* *η*_*j*_ ∈ {0, 1} *and the constraint* $\frac{L_{j}}{\sum_{i=1}^{N_{r}}\alpha_{ij}(C_{ij}-a_{j})-b_{j}}-DL_{j}\leq M\eta_{j}$, *where* *M* *is a value greater than all the values for the argument of* *H*. *Thus the optimization problem of minimizing the function* *f* *can be reformulated as the next optimization problem where the variable are now* *η*_*j*_ *and* *α*_*ij*_:

$$\begin{array}{c} \begin{array}{cc} & \underset{\eta_{j},\alpha_{ij}}{minimize}\sum_{j=1}^{N_{t}}\eta_{j}\\ & subject\;to\\ & \frac{L_{j}}{\sum_{i=1}^{N_{r}}\alpha_{ij}\left(C_{ij}-a_{j}\right)-b_{j}}-DL_{j}\leq M\eta_{j}\;\text{for}\;\text{all}\;1\leq j\leq N_{t} \end{array} \end{array}$$ (18)

*In the light of the exposed considerations*, *it is clear that the multi-robot task allocation problem under consideration can be seen as an optimization problem which fits the characteristics of the* Problem (18). *Indeed*, *the fact that any* *η*_*j*_ *takes the value 0 can be interpreted as the coalition of robots allocated in task* *t*_*j*_ *can carry out the task before its deadline (of course* *η*_*j*_ = 1 *can be interpreted dually)*. *Moreover*, *each utility function* *U*_*j*_ *can be expressed in terms of the* *H* *functions as follows*:

$$\begin{array}{c} U_{j}\left(\tau \right)=U_{max_{j}}H\left(\tau -DL_{j}\right), \end{array}$$ (19)

*where* *τ* *is the amount of time required to finish the task* *t*_*j*_. *This expression can be rewritten to add robots’ capabilities*, *task’s characteristics and interference*, *as follows*:

$$\begin{array}{c} U_{j}\left(\frac{L_{j}}{\sum_{i=1}^{N_{r}}\alpha_{ij}\left(C_{ij}-a_{j}\right)-b_{j}}\right)=U_{max_{j}}H\left(\frac{L_{j}}{\sum_{i=1}^{N_{r}}\alpha_{ij}\left(C_{ij}-a_{j}\right)-b_{j}}-DL_{j}\right). \end{array}$$ (20)

*After taking into account the information provided by the utility functions* *U*_*j*_ *and the interference effect*, Problem (18) *becomes the following optimization problem*:

$$\begin{array}{c} \begin{array}{cc} & \underset{\eta_{j},\alpha_{ij}}{minimize\;}\sum_{j=1}^{N_{t}}U_{max_{j}}\eta_{j}\\ & subject\;to\\ & \eta_{j}m-\sum_{i=1}^{N_{r}}\alpha_{ij}\left(C_{ij}-a_{j}\right)\leq -\left(\frac{L_{j}}{DL_{j}}+b_{j}\right)\;\text{for}\;\text{all}\;j=1,\ldots ,N_{t}\\ & \sum_{j=1}^{N_{t}}\alpha_{ij}\leq 1\;\text{for}\;\text{all}\;i=1,\ldots ,N_{r} \end{array} \end{array},$$ (21)

*where* $m=\max_{j}\left\{-\frac{L_{j}}{\sum_{i=1}^{N_{r}}\alpha_{ij}\left(a_{j}-1\right)+b_{j}}\right\}$ *for all* *j* = 1, …, *N*_*t*_. *Consequently*, *when the utility functions meet the characteristics of the Case 3*, *the optimization problem matches up with a binary linear programming problem*.

Table 1 summarizes the aforementioned cases, assuming that the interference function *I*_*g*_ is linear. In spite of most MRTA cases can be addressed as linear (binary-integer) optimization problems they still require too much time to be solved in dynamic environments, especially when there are a large number of tasks or robots. As pointed in section about previous work, many approaches have tried to give a good enough solution with a reasonable amount of time: swarm intelligence, auction approaches, and so on, but only few of them take into account task with deadlines. Inspired by the preceding fact and by the exposed example, a new auction-like algorithm to address MRTA-IA problems is introduced in the next section.

**Table 1:** Summary of the complexity regarding the utility function, where BINLP is binary integer nonlinear programming problem; BILP denotes integer linear programing problem problem; LP is linear programing problem.

**Case 1**	BINLP
**Case 2**	BILP
**Case 3**	BILP

## Task Allocation: Double Round Auction for tasks with deadline

In this section we first overview an auction method, called from now on Simple Double Round Auction (SDRA), that was previously proposed by the first two authors in [3]. The experimental results showed that the aforementioned method outperforms the current auction approaches. Furthermore, to our best knowledge, this is the only method that addresses coalition formation for tasks with deadlines. Then, in the light of the experimental results and motivated by the problem formalization explained in the last section, a new auction method, called Multiple Objectives Double Round Auction (MDRA), is proposed for the first time.

### Simple Double Round Auction (SDRA)

The SDRA algorithm splits the classical auction process into two steps: *auction for a task* and *auction for a robot*. In each task there is a robot, called the *task leader*, that will manage an auction process. Thus, several auction processes can be executed in parallel; one for each task. Each task must have a single leader which is the robot that handles the coalition or work group, that is, it will be the auctioneer. In the initial stage, each robot is looking for a task. When a robot finds a new task, it tries to lead it. As there can only be one leader per task, the candidate will execute the leadership request process to ensure at most one leader per group.

If a robot is promoted to the leadership of the task *t*_*j*_, it creates a coalition of robots, if necessary. In that case, the leader must decide the optimum group size and which robots are part of it. To take this decision, the leader uses the first step of the SDRA, called *auction for a task*. In this process the leader is the auctioneer and the other robots bid using their work capacities, *C*_*ij*_. After a given time, the leader selects the robots with the highest work capacity using a greedy algorithm until it detects that, even if the next best robot is added to the group, the task utility function *U*_*j*_ is not improved. Following the new formulation, if *U*_*j*_ ∘ *β*_*j*_ function meets the constraints of the Case 2 detailed in Section Case studies, the task leader selects the best robots until this condition is verified:

$$\begin{array}{c} U_{j}\left(\beta_{j}\left(g_{B}\right)\right)\geq U_{j}\left(\beta_{j}\left(g_{B}^{\prime }\right)\right), \end{array}$$ (22)

where *g*_*B*_ stands for the group of best robots already selected and $g_{B}^{\prime }=g_{B}\cup \left\{r_{nmax}\right\}$ where *r*_*nmax*_ is the next best robot. If *U*_*j*_ is a hard-deadline function (see Case 3), the task leader stops selecting new robots when it detects that the group is able to reach the task deadline, that is until *β*_*j*_(*g*_*B*_) ≤ *DL*_*j*_.

Furthermore, the leader does not include any selected robot in its group until it receives the confirmation from these candidates. This confirmation is generated during the *auction for a robot* round. In this round, to get the selected robots, the leader bids for them sending the expected utility of the task. Therefore, now the leader becomes the bidder and the selected robots are the auctioneers. When a robot receives a bid from a leader, it waits a certain time for more bids from other leaders. After this time, the robot selects the coalitions with the greatest bid and it becomes a member sending a confirmation message to the corresponding leader.

The interference factor *I*_*gj*_, required to calculate the expected execution time, (see Eqs (4) and (5)), was estimated using a Support Vector Regression method (SVR). In the experimental results section, we will explain in a more detailed way how the interference parameters have been estimated.

### Multiple Objectives Double Round Auction (MDRA)

When applying the SDRA method, the *auction for a robot* round only used the utility sent by the leader and did not take into account the individual capacity of the robots. The new method, called Multiple Objectives Double Round Auction (MDRA), uses both the individual and the leader’s utility during the *auction for a robot* round in order to improve the system performance. The other two steps of the auction process: leader’s selection and *auction for a task*, are not modified with respect to SDRA.

In the MDRA method, as in SDRA, the robots selected by the leaders receive the expected utility of the tasks, denoted by *B* = {(*t*_1_, *b*_1_), …, (*t*_*n*_, *b*_*n*_)}, where $b_{i}\in R^{+}$ represents the utility of the group that attempts to execute the task *t*_*i*_ ∈ *T*. Let (*t*_*best*_, *b*_*best*_) be the tuple in *B* with highest utility; and denote by *B*_*bests*_ = {(*t*_*i*_, *b*_*i*_) ∈ *B*: *b*_*i*_ ≥ λ_*B*_ ⋅ *b*_*best*_} where λ_*B*_ ∈ [0, 1]. The robot will select the task from *B*_*bests*_ for which it has the highest capacity. Algorithm 1 specifies the steps executed by a non-leader robot to implement the *auction for a robot* stage, where *TIME*_*BID*_*ACCEPTED* stands for the amount of time that the robot waits for leaders bids (*AWARD* messages). After this time, the robot selects the coalition with the greatest bid and it becomes a member sending a confirmation message (*ROBOT*_*ALIVE*) to the corresponding leader. If the leader doesn’t receive any answer from a robot or receives a reject message (*REFUSE*), it removes that robot from its list of selected robots. On the other hand, a robot is definitely added to the group, when the leader receives a confirmation message from it. The λ_*B*_ value is inspired by the selection factor used in [13], but it is now applied in an auction process with tasks’ deadlines.

**Algorithmn 1** *Auction for a robot* round executed in each no leader robot

**Require**: λ_*B*_ ∈ [0, 1]

**Require**: *time* current time

1: **if** *AWARD* message, (*t*_0_, *b*_0_), from a leader **then**

2:  *B* ← (*t*_0_, *b*_0_)

3:  *time*_*init*_ ← *time*

4:  **while** *time* − *time*_*init*_ ≤ *TIME*_*BID*_*ACCEPTED* **do**

5:   **if** *AWARD* message, (*t*_*j*_, *b*_*j*_), from a leader **then**

6:    *B* ← *B* ⋃ (*t*_*j*_, *b*_*j*_)

7:    *j* ← *j* + 1

8:   **end if**

9:  **end while**

10:  (*t*_*best*_, *b*_*best*_) tuple in *B* with the maximum bit value

11:  *B*_*bests*_ = {(*t*_*i*_, *b*_*i*_) ∈ *B*: *b*_*i*_ ≥ λ_*B*_ ⋅ *b*_*best*_}

12:  *t*_*sel*_ ← task from *B*_*bests*_ with the highest robot’s capacity

13:  Send a *ROBOT*_*ALIVE* message to the leader of *t*_*sel*_

14:  **for all** *t*_*j*_ in *B* and *t*_*i*_ ≠ *t*_*sel*_ **do**

15:   Send *REFUSE* message to the leader of *t*_*j*_

16:  **end for**

17: **end if**

## Experimental results

In this section we detail the experiments executed in order to analyze the behaviour of the auction algorithms, comparing them to the optimal solution given by the optimization problems of Section Coalition formation complexity analysis. Firstly, we will explain the experiments carried out with a realistic (physical) simulator called RoboCoT (see [3]) in order to obtain the *I* function parameters. From the function *I*, new experiments have been carried out using Matlab (Version R2014a and Optimization Toolbox version 7). The solutions for the optimization, Problems 15 and 21, have been obtained executing the optimal linear programming-based branch-and-bound algorithm together with a simplex method [48].

Besides the approaches already explained, another simple method, called greedy selection, has been implemented. In this method each robot selects the best task from its point of view, in a selfish mode. Thus, a robot *r*_*i*_ selects the task *t*_*sel*_ such that, $t_{sel}=\underset{j}{max}\left(C_{ij}\right)$. The robots do not execute any auction algorithm nor make any prediction about the interference.

### Experiments with physical robots: Interference function

**Fig 2:**
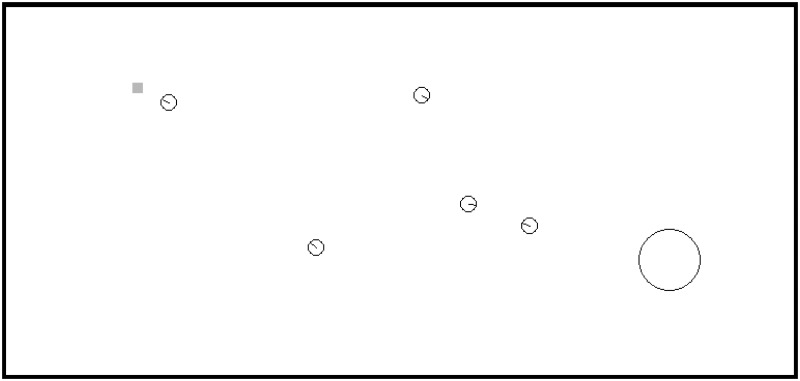
Execution example of a RoboCoT experiment to calculate the parameters of the interference function *I*.

First, we describe how the parameters of the interference function (Eq 14) have been estimated. In a previous work [49], the first two authors exposed how, for foraging-like tasks (see Section Example: Foraging with tasks’ deadline), the interference can be modelled as a set of polynomial functions that only depends on the number of robots and on the distance between the delivery point and the object. To get the parameters of the polynomial functions the following experiment in the realistic simulator RoboCoT was carried out: several robots must transport a unique object, formed by transportable bits, and the total weight transported by the robots after 40,000 time units is calculated. Seven different distances between object and delivery point were tested and the parameters of *I* were fitted for each one of them (see Table 2). Fig 2 shows an snapshot of one of these executions, where the little square represents the object, the big circle the delivery point and the little circles are the robots. These results were also validated with other well-known simulator called Player/Stage [50]. Although all the interference values in this paper relate to physical simulations, the algorithms were also implemented on four real Pioneer-3DX. Videos with some executions can be watched in [51] and a more detailed description of these experiments can be found in [49].

**Table 2:** Parameter *a*_*j*_ of the linear interference function.

Distance	*a*_*j*_ ⋅ 10^3^
D1 = 138	10.3428
D2 = 184	6.1682
D3 = 247	3.4728
D4 = 282	2.8746
D5 = 329	1.9278
D6 = 359	1.7190
D7 = 400	1.3657

From the results of the aforesaid simulations, it can be seen that the maximum utility of the group (the maximum of Eq (5)) is reached when the number of robots is equal to or lower than 30. Thanks to the condition given by Eq (22), the maximum number of robots in a coalition created by the auction algorithms is 30. Although in the original paper [49] the polynomials have a degree equals to two, if *n*_*g*_ < 30 the *I*_*g*_ can be fitted by a linear polynomial function with a very low error, meeting all the conditions of the cases exposed in section about the formalization (Cases 2 and 3). The parameter *b*_*j*_ of Eq (14) was set to 0 because it is assumed that a coalition with a single robot does not have physical interference. Table 2 shows the interference linear function parameter *a*_*j*_ that minimizes the square error for each distance tested.

### Experiments with soft-deadline utility functions

The first set of experiments assume that each *U*_*j*_ ∘ *β*_*j*_ is linear and it meets the condition of Case 2. Therefore the problem to solve is Problem (15). The characteristics of the experiments are the following: The number of robots (*N*_*r*_) varies from 2 to 30 times the number of objects (*N*_*t*_). There are 5 different types of objects and each robot has a different load capacity depending on the object type. In order to apply the parameters of Table 2, the distances always fall into the bounds described in above section. In addition, robots’ work capacity *C*_*ij*_ depends on the type of task and on its velocity, as is pointed out by the authors in [3]. The main goal of these experiments is to test the new MDRA algorithm with a very large number of robots. It is worth to point out that when *N*_*t*_ = 15 there are up to 450 robots, and if *N*_*t*_ = 30 then this number is increased up to 900 robots. Each experiment has been performed 500 times using a uniform random distribution to generate the position of the objects, their weights, the load capacity of the robots and their velocities. S1 Table shows the parameters of the uniform random variables for each one of these characteristics. In order to keep the robots as simple as possible, the robots use their load capacities for selecting the best task in the Greedy strategy.

**Fig 3:**
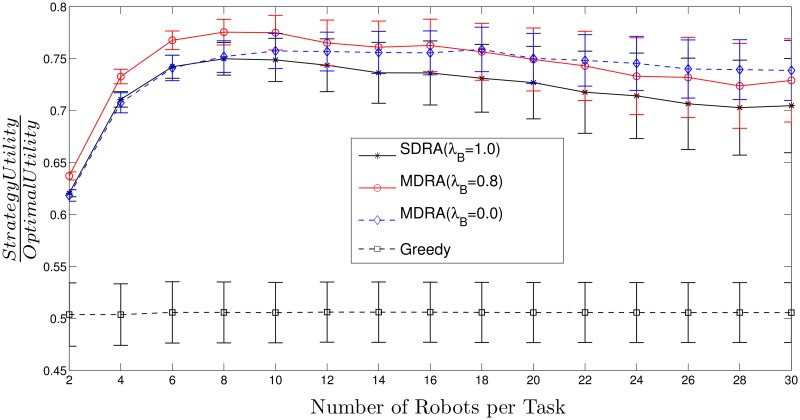
Ratio between utility obtained with MDRA and the utility of the optimal strategy with *N*_*t*_ = 15. The bars stand for the standard deviation (*σ*^2^).

**Fig 4:**
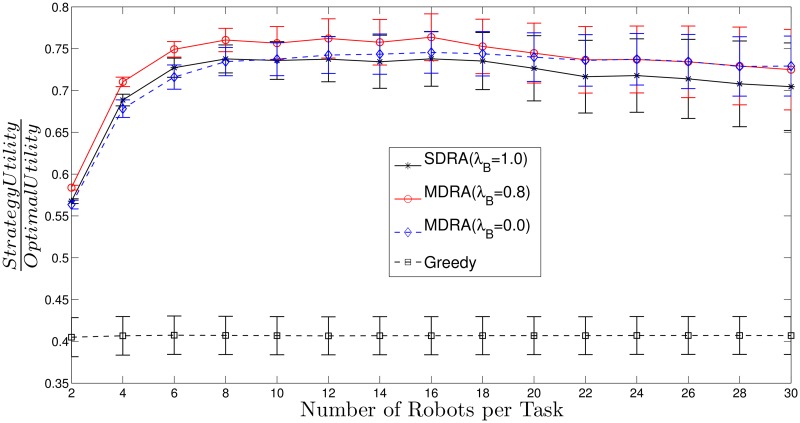
Ratio between utility obtained with MDRA and the utility of the optimal strategy with *N*_*t*_ = 30. The bars stand for the standard deviation (*σ*^2^).

Fig 3 (*N*_*t*_ = 15) and Fig 4 (*N*_*t*_ = 30) show the utility per robot obtained by different strategies (SDRA, MDRA and greedy) divided by the utility per robot obtained with the optimal method (see S2 and S3 Tables). For all these results, utility per robot stands for the mean of the total obtained utility divided by the number of robots. Thus, higher values stand for results closer to the optimal. The X-axis shows the ratio between the number of robots and the total number of tasks and the bars stand for the standard deviation (*σ*^2^). As can be seen, the total utility obtained with MDRA utilities is close to the optimal and the better results (higher values) are obtained when the number of robots per task is 8. With more than 8 robots per task, the results stay constant or show a slight decrease. For some cases, MDRA utility reaches more than the 75% of the optimal utility and from around 14 robots per task this value keeps almost constant. If, instead of the mean, it is calculated the median, the MDRA utility reaches more than an 80% of the optimal (see S3 Table). The Greedy strategy provides the worst results, always lower than a 55% of the optimal utility. In most cases, the new MDRA algorithm gives the best results when λ_*B*_ = 0.8 and outperforms the SDRA. It should be recalled that the tested environments are very heterogeneous and, therefore, this issue increases the *σ*^2^ values of the experimental results.

**Fig 5:**
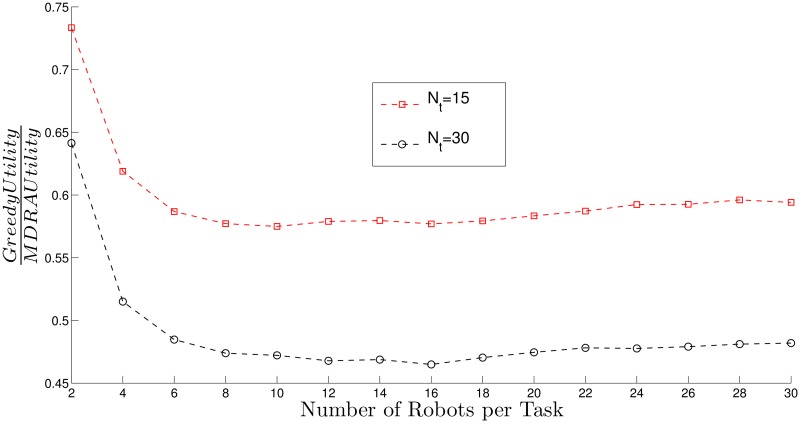
Ratio between greedy utility and MDRA (λ_*B*_ = 0.8).

Fig 5 shows the ratio between utility per robot using greedy algorithm and MDRA with λ_*B*_ = 0.8, similar results are obtained for other values of λ_*B*_. The experiments demonstrate that the higher number of robots the lower greedy performance with respect to MDRA and hence, the new auction algorithm improves the results compared to a fully distributed approach. The standard deviation of these experiments can be obtained from the information of the Figs 3 and 4.

**Fig 6:**
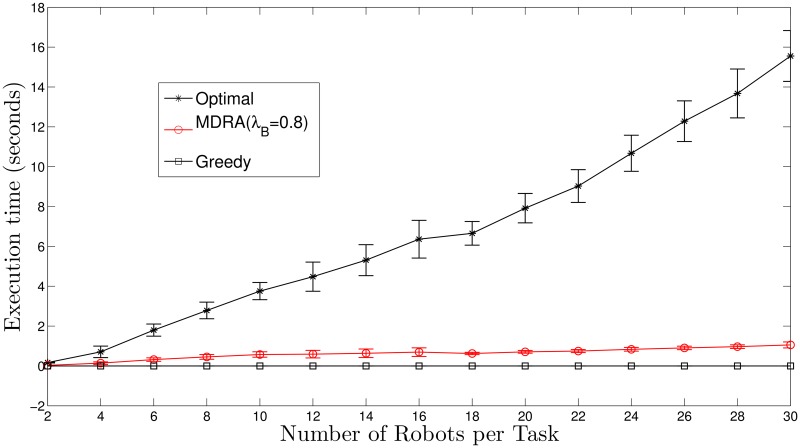
Execution time for soft deadline experiments and *N*_*t*_ = 30. The bars stand for the standard deviation (*σ*^2^).

As can be seen in Fig 6 (see also S4 Table), the optimal algorithm needs much more execution time to give a solution than MDRA with λ_*B*_ = 0.8 or greedy algorithm and it clearly grows with the number of robots. Moreover, the MDRA algorithm increases very slowly its execution time with respect to the number of robots. Therefore, it can be concluded that the results provided by the new MDRA method are pretty close to the optimal results with much less execution time and it outperforms the SDRA approach.

### Experiments with hard-deadline utility functions

This section describes the experiments carried out with hard-deadline utility functions and lineal interference, that is, Case 3 described in Section Case studies. Therefore, the problem to solve is the same as Problem (21). In order solve it numerically, we must find coalitions with a performance (amount of work carried out per time unit) greater or equal than the performance needed to finish the task before the deadline, instead of the execution time. Notice that both problems are equivalent and, therefore, the resulting solutions are exactly the same. Moreover the new problem has the same form as the Problem 21 but with $m=\min_{j}\left(-\frac{L_{j}}{DL_{j}}-b_{j}\right)$, for all *j* = 1, …, *N*_*t*_. The new *m* value is simpler than the original one and avoids division by zero problems.

As for soft-deadline experiments, each experiment has been performed 500 times using a uniform random distribution to generate the position of each object, its weight and deadline, the load capacity of the robots and their velocities. As the scalability of the system has already been demonstrated in the previous section, all the simulations have been performed with 4 tasks (*N*_*t*_ = 4) and the robots always use their expected capacities for selecting the best robot (Greedy) and for bidding (SDRA or MDRA).

**Fig 7:**
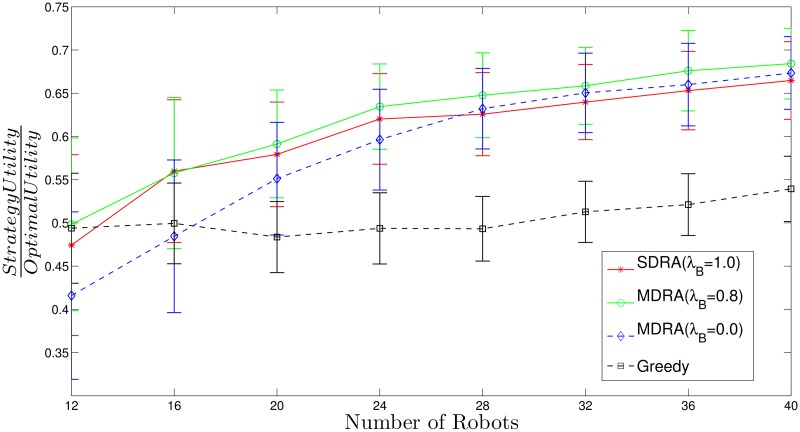
Ratio between auction or greedy utility and optimal utility with hard-deadline. The bars stand for the standard deviation (*σ*^2^).

**Fig 8:**
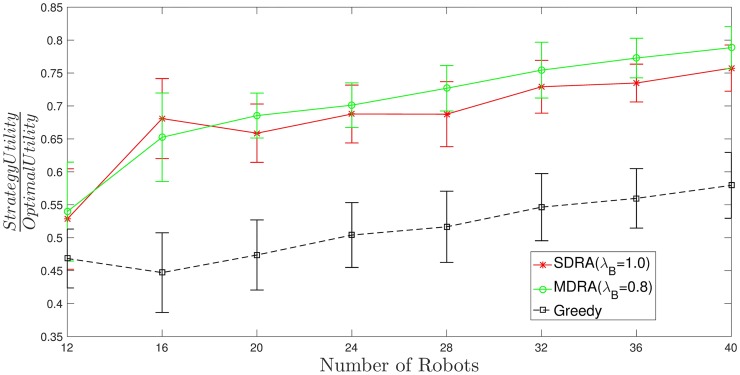
Ratio between auction or greedy utility and optimal utility with hard-deadline using the 50 experiments with the highest tasks’ deadline values. The bars stand for the standard deviation (*σ*^2^).

Fig 7 (see also S5 Table) shows the utility per robot for different strategies (SDRA, MDRA and greedy) divided by the utility per robot obtained with the optimal method, where the bars stand for the standard deviation *σ*^2^. As can be seen, the higher number of robots the higher value of this ratio for MDRA, which means that the MDRA results are getting closer to the optimal. Despite of the utility per robot provided by MDRA decreases compared to the soft-deadline case, it reaches values near the 70% of the optimal (ratio = 0.7). This must be considered a very good result, specially tanking into account that the complexity of the problem is dramatically increased, which implies that the MATLAB solver takes order of minutes to give a results while MDRA keeps bounded on the order of seconds. In all the cases, the MDRA outperforms the SDRA when λ_*B*_ = 0.8, as happened for soft-deadline utility function experiments. So the optimal value of λ_*B*_ seems to be very stable for any environment or utility function. Furthermore, the greedy algorithm keeps very far from the optimal and even from the auction approaches. Finally, the standard deviation decreases regarding the number of robots. The overlap in the standard deviation bars is due to the great heterogeneity of the carried out experiments. In order to cover a broad spectrum of situations, the standard deviations of the uniform random variables used to generate each experiment are very high, as can be seen in S1 Table. If we focus on a specific set of experiments, the aforementioned overlap is reduced. For example, Fig 8 (see also S6 Table) shows the ratio between auction or greedy utility and optimal utility with hard-deadline over the 50 experiments with the highest tasks’ deadline values. For the sake of simplicity this figure only shows the MDRA results with λ_*B*_ = 0.8. As can be seen, the overlap between the standard deviation of SDRA and MDRA is reduced regarding Fig 7. In order to address these problems, future work will analyze the impact of the environment configuration on the system performance.

**Fig 9:**
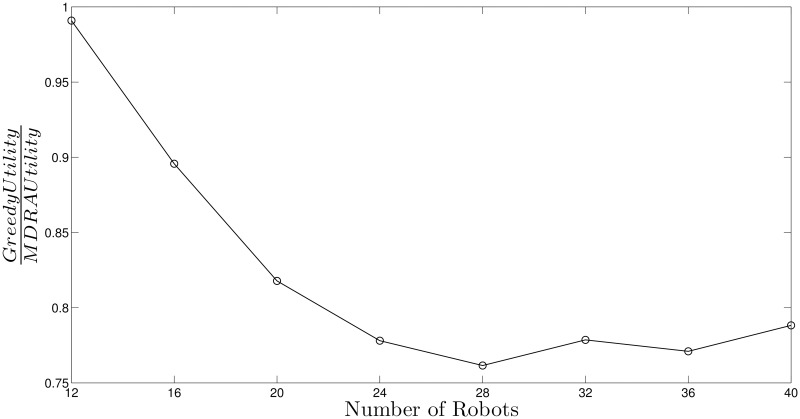
Ratio between greedy utility and MDRA (λ_*B*_ = 0.8) for hard-deadline tasks.

As in previous section, Fig 9 shows the ratio between utility per robot using greedy algorithm and MDRA with λ_*B*_ = 0.8, similar results are obtained for other values of λ_*B*_. The results demonstrate how the performance of the greedy algorithm decreases regarding the number of robots. The standard deviation of these experiments can be obtained from the information of Fig 7.

## Conclusions and future work

This paper has presented the first formal study about the interference impact on the complexity of the multi-robot coalition formation problem to allocate tasks with deadlines. The formalization of the MRTA problem shows that, on the one hand the interference can be modelled by a linear function and, on the other hand, the optimal solution can be obtained solving an integer linear problem which depends on the utility function characteristics.

As a secondary contribution of this paper, a new auction-like method, called Multiple Objectives Double Round Auction (MDRA), is also presented. A large number of experiments, with both hard and soft-deadline utility functions, has been carried out in order to compare this new approach to the optimal solutions. The results show that MDRA outperforms previous auction methods in the literature and can reach between a 70% (for the hard-deadline utility functions) and more than an 80% (for the soft-deadline utility functions) of the optimal with a very low computational time.

Regarding the aspects to improve this work in the near future, our research is now focused on defining new models for the interference and for measurable errors like: robot’s kinematic errors, localization errors, and so on. As pointed out in this paper, other authors propose swarm-like strategies to face up the physical interaction between thousands of robots. Despite our paper does not address swarm issues, the proposed MRTA strategies can be seen as a previous stage before applying lower level swarm-like methodologies. Thus, from the results of these authors (see [45]), we will study how the physical interaction impacts on our MRTA algorithms. Moreover, non-linear interference models, that would need to be addressed with genetic algorithms, and methods for optimizing several goals simultaneously are under consideration. A preemptive auction method, that is, a method that allows the exchange of robots between working groups, is also under study. Finally, we will study how the environments characteristics impact on the system performance.

## Supporting Information

S1 Table Parameters of the uniform random variables (*U*(*a*, *b*)) used to generate the experiments. (PDF)

S2 Table Mean, standard deviation and median for experiments with soft deadline and *N*_*t*_ = 15. These results show the ratio between the utility obtained with MDRA and the utility of the optimal strategy.
(PDF)

S3 Table Mean, standard deviation and median for experiments with soft deadline and *N*_*t*_ = 30. These results show the ratio between the utility obtained with MDRA and the utility of the optimal strategy.
(PDF)

S4 Table Mean, standard deviation and median of the execution time with soft deadline and *N*_*t*_ = 30. (PDF)

S5 Table Mean, standard deviation and median of the execution with hard deadline. These results show the ratio between the utility obtained with MDRA and the utility of the optimal strategy.
(PDF)

S6 Table Mean, standard deviation and median of the execution with hard deadline using the 50 environments with the highest deadline values. These results show the ratio between the utility obtained with MDRA and the utility of the optimal strategy.
(PDF)
