# Supplementary material for: Multi-Robot Coalitions Formation with Deadlines: Complexity Analysis and Solutions
Source: PLoS One. 2017 Jan 24;12(1):e0170659. doi: 10.1371/journal.pone.0170659 (PMC5261615; doi:10.1371/journal.pone.0170659)
Supplement: S1 Table — (PDF) [file pone.0170659.s001.pdf]

Parameters of the uniform random variables (U(a,b)) used to generate the experiments

Objects' Characteristics				
**Variable**	**a**	**b**	**Mean**	**Variance**
Position (x,y)	-283	283	0,00	47,17
Weight	10	30	20,00	33,33
Utility	100	200	150,00	833,30
Deadline	50	400	225,00	10208,33

Robots' Characteristics				
**Variable**	**a**	**b**	**Mean**	**Variance**
Load Capacity	1	5	3,00	1,33
Velocity	1	5	3,00	1,33
