# Supplementary material for: Multi-Robot Coalitions Formation with Deadlines: Complexity Analysis and Solutions
Source: PLoS One. 2017 Jan 24;12(1):e0170659. doi: 10.1371/journal.pone.0170659 (PMC5261615; doi:10.1371/journal.pone.0170659)
Supplement: S2 Table — These results show the ratio between the utility obtained with MDRA and the utility of the optimal strategy. (PDF) [file pone.0170659.s002.pdf]

Results of Experiments with Soft Deadline and 15 tasks.
Ratio between utility obtained with MDRA and the utility of the optimal strategy

Mean							
Robots per task	λ_B=1.0	λ_B=0.8	λ_B=0.6	λ_B=0.4	λ_B=0.2	λ_B=0.0	Greedy
2	0,6205	0,6372	0,6395	0,6304	0,6194	0,6183	0,5038
4	0,7108	0,7328	0,7391	0,7231	0,7103	0,7075	0,5038
6	0,7422	0,7676	0,7711	0,7601	0,7450	0,7411	0,5059
8	0,7499	0,7755	0,7780	0,7688	0,7571	0,7520	0,5059
10	0,7487	0,7750	0,7759	0,7707	0,7605	0,7574	0,5057
12	0,7436	0,7651	0,7713	0,7671	0,7589	0,7568	0,5062
14	0,7364	0,7610	0,7675	0,7667	0,7595	0,7560	0,5061
16	0,7362	0,7627	0,7686	0,7647	0,7592	0,7555	0,5061
18	0,7311	0,7566	0,7670	0,7673	0,7622	0,7589	0,5059
20	0,7270	0,7492	0,7582	0,7569	0,7523	0,7502	0,5057
22	0,7177	0,7430	0,7516	0,7528	0,7516	0,7483	0,5058
24	0,7142	0,7331	0,7449	0,7468	0,7459	0,7454	0,5057
26	0,7065	0,7319	0,7394	0,7411	0,7404	0,7400	0,5057
28	0,7029	0,7238	0,7351	0,7429	0,7432	0,7394	0,5057
30	0,7047	0,7290	0,7369	0,7402	0,7430	0,7387	0,5057

Standard Deviation (δ^2)							
Robots per task	λ_B=1.0	λ_B=0.8	λ_B=0.6	λ_B=0.4	λ_B=0.2	λ_B=0.0	Greedy
2	0,0035	0,0039	0,0042	0,0048	0,0054	0,0055	0,0304
4	0,0075	0,0070	0,0066	0,0083	0,0098	0,0096	0,0296
6	0,0111	0,0089	0,0089	0,0107	0,0128	0,0123	0,0295
8	0,0157	0,0123	0,0136	0,0147	0,0157	0,0152	0,0293
10	0,0208	0,0167	0,0171	0,0173	0,0178	0,0169	0,0291
12	0,0254	0,0219	0,0202	0,0200	0,0199	0,0186	0,0290
14	0,0293	0,0252	0,0222	0,0210	0,0210	0,0202	0,0290
16	0,0308	0,0252	0,0235	0,0229	0,0222	0,0211	0,0289
18	0,0326	0,0274	0,0245	0,0232	0,0225	0,0214	0,0289
20	0,0349	0,0303	0,0278	0,0272	0,0260	0,0240	0,0290
22	0,0395	0,0333	0,0310	0,0292	0,0263	0,0248	0,0289
24	0,0411	0,0369	0,0327	0,0294	0,0281	0,0260	0,0289
26	0,0439	0,0386	0,0351	0,0311	0,0302	0,0280	0,0288
28	0,0457	0,0408	0,0370	0,0318	0,0299	0,0288	0,0288
30	0,0453	0,0400	0,0365	0,0328	0,0305	0,0289	0,0288

Median							
Robots per task	λ_B=1.0	λ_B=0.8	λ_B=0.6	λ_B=0.4	λ_B=0.2	λ_B=0.0	Greedy
2	0,6153	0,6353	0,6403	0,6336	0,6218	0,6199	0,5089
4	0,7283	0,7524	0,7578	0,7477	0,7370	0,7297	0,5151
6	0,7660	0,7950	0,8036	0,7943	0,7736	0,7664	0,5104
8	0,7810	0,8100	0,8175	0,8056	0,7865	0,7807	0,5159
10	0,7890	0,8156	0,8242	0,8143	0,7879	0,7799	0,5125
12	0,7929	0,8144	0,8180	0,8024	0,7853	0,7803	0,5140
14	0,7888	0,8149	0,8147	0,7912	0,7768	0,7727	0,5122
16	0,7913	0,8142	0,8165	0,7961	0,7795	0,7696	0,5112
18	0,7861	0,8076	0,8070	0,8010	0,7849	0,7719	0,5095

20	0,7861	0,8018	0,8024	0,7899	0,7733	0,7651	0,5079
22	0,7798	0,8002	0,7981	0,7843	0,7751	0,7678	0,5077
24	0,7691	0,7878	0,7849	0,7693	0,7588	0,7608	0,5079
26	0,7590	0,7852	0,7827	0,7571	0,7570	0,7517	0,5079
28	0,7602	0,7659	0,7651	0,7601	0,7518	0,7425	0,5097
30	0,7568	0,7680	0,7662	0,7549	0,7553	0,7420	0,5081
