# Supplementary material for: Multi-Robot Coalitions Formation with Deadlines: Complexity Analysis and Solutions
Source: PLoS One. 2017 Jan 24;12(1):e0170659. doi: 10.1371/journal.pone.0170659 (PMC5261615; doi:10.1371/journal.pone.0170659)
Supplement: S3 Table — These results show the ratio between the utility obtained with MDRA and the utility of the optimal strategy. (PDF) [file pone.0170659.s003.pdf]

Results of Experiments with Soft Deadline and 30 tasks.

Ratio between utility obtained with MDRA and the utility of the optimal strategy

Mean							
Robots per task	λ_B=1.0	λ_B=0.8	λ_B=0.6	λ_B=0.4	λ_B=0.2	λ_B=0.0	Greedy
2	0,5676	0,5838	0,5862	0,5754	0,5648	0,5636	0,4050
4	0,6886	0,7103	0,7106	0,6960	0,6816	0,6783	0,4066
6	0,7273	0,7492	0,7474	0,7327	0,7208	0,7160	0,4074
8	0,7377	0,7604	0,7629	0,7500	0,7405	0,7346	0,4071
10	0,7359	0,7566	0,7582	0,7493	0,7399	0,7375	0,4068
12	0,7375	0,7623	0,7631	0,7537	0,7452	0,7424	0,4066
14	0,7344	0,7577	0,7591	0,7503	0,7466	0,7435	0,4067
16	0,7378	0,7637	0,7646	0,7551	0,7476	0,7455	0,4067
18	0,7352	0,7527	0,7591	0,7497	0,7453	0,7439	0,4068
20	0,7265	0,7447	0,7493	0,7435	0,7411	0,7399	0,4068
22	0,7165	0,7367	0,7453	0,7382	0,7378	0,7360	0,4068
24	0,7179	0,7371	0,7451	0,7406	0,7392	0,7374	0,4070
26	0,7139	0,7343	0,7400	0,7332	0,7341	0,7346	0,4070
28	0,7079	0,7293	0,7352	0,7299	0,7281	0,7288	0,4070
30	0,7045	0,7249	0,7328	0,7312	0,7300	0,7292	0,4069

Standard Deviation (δ^2)							
Robots per task	λ_B=1.0	λ_B=0.8	λ_B=0.6	λ_B=0.4	λ_B=0.2	λ_B=0.0	Greedy
2	0,0028	0,0029	0,0032	0,0044	0,0052	0,0052	0,0234
4	0,0070	0,0057	0,0065	0,0092	0,0106	0,0106	0,0231
6	0,0115	0,0093	0,0106	0,0138	0,0149	0,0145	0,0230
8	0,0167	0,0140	0,0136	0,0162	0,0171	0,0169	0,0229
10	0,0225	0,0198	0,0191	0,0203	0,0203	0,0197	0,0229
12	0,0270	0,0235	0,0228	0,0235	0,0229	0,0220	0,0228
14	0,0318	0,0273	0,0263	0,0270	0,0246	0,0241	0,0228
16	0,0327	0,0280	0,0262	0,0265	0,0259	0,0248	0,0228
18	0,0341	0,0326	0,0295	0,0301	0,0280	0,0266	0,0228
20	0,0390	0,0358	0,0339	0,0330	0,0304	0,0291	0,0227
22	0,0435	0,0398	0,0359	0,0347	0,0321	0,0308	0,0226
24	0,0439	0,0400	0,0361	0,0342	0,0315	0,0308	0,0227
26	0,0473	0,0428	0,0399	0,0376	0,0346	0,0324	0,0227
28	0,0512	0,0465	0,0426	0,0396	0,0370	0,0355	0,0226
30	0,0524	0,0481	0,0436	0,0390	0,0371	0,0359	0,0226

Median							
Robots per task	λ_B=1.0	λ_B=0.8	λ_B=0.6	λ_B=0.4	λ_B=0.2	λ_B=0.0	Greedy
2	0,5687	0,5840	0,5866	0,5807	0,5726	0,5712	0,3991
4	0,7082	0,7257	0,7320	0,7257	0,7118	0,7040	0,3971
6	0,7519	0,7779	0,7816	0,7722	0,7562	0,7468	0,3982

8	0,7723	0,8026	0,8052	0,7933	0,7757	0,7668	0,3965
10	0,7775	0,8062	0,8046	0,7856	0,7770	0,7686	0,3983
12	0,7855	0,8109	0,8133	0,7977	0,7855	0,7778	0,3968
14	0,7928	0,8154	0,8168	0,7986	0,7803	0,7772	0,3965
16	0,7934	0,8218	0,8179	0,8000	0,7818	0,7708	0,3974
18	0,7937	0,8133	0,8140	0,8003	0,7723	0,7719	0,3987
20	0,7904	0,8109	0,8097	0,7864	0,7692	0,7717	0,3980
22	0,7873	0,8056	0,7990	0,7846	0,7769	0,7664	0,3956
24	0,7914	0,8066	0,7928	0,7815	0,7703	0,7637	0,3973
26	0,7928	0,8076	0,8036	0,7800	0,7772	0,7647	0,3986
28	0,7880	0,8046	0,7895	0,7744	0,7664	0,7604	0,3979
30	0,7874	0,8052	0,7951	0,7794	0,7683	0,7640	0,3957
