# Supplementary material for: Multi-Robot Coalitions Formation with Deadlines: Complexity Analysis and Solutions
Source: PLoS One. 2017 Jan 24;12(1):e0170659. doi: 10.1371/journal.pone.0170659 (PMC5261615; doi:10.1371/journal.pone.0170659)
Supplement: S4 Table — (PDF) [file pone.0170659.s004.pdf]

Mean, standard deviation and median of the execution time with soft deadline and 30 robots.

Mean								
Robots per task	Optimal	λ_B=1.0	λ_B=0.8	λ_B=0.6	λ_B=0.4	λ_B=0.2	λ_B=0.0	Greedy
2	0,1679	0,0314	0,0312	0,0311	0,0312	0,0312	0,0312	0,0002
4	0,7068	0,1465	0,1427	0,1395	0,1333	0,1321	0,1311	0,0004
6	1,7985	0,3387	0,3188	0,3029	0,3007	0,2969	0,2921	0,0007
8	2,7854	0,4780	0,4547	0,4401	0,4363	0,4199	0,4096	0,0008
10	3,7553	0,6146	0,5739	0,5571	0,5381	0,5324	0,5418	0,0009
12	4,4794	0,6228	0,5913	0,5710	0,5604	0,5532	0,5517	0,0009
14	5,3095	0,6766	0,6368	0,6166	0,6031	0,6012	0,5822	0,0008
16	6,3608	0,7337	0,6939	0,6724	0,6589	0,6503	0,6417	0,0009
18	6,6566	0,6260	0,6258	0,6258	0,6241	0,6264	0,6261	0,0008
20	7,9187	0,7080	0,7065	0,7065	0,7031	0,7032	0,7047	0,0008
22	9,0280	0,7480	0,7455	0,7437	0,7426	0,7429	0,7432	0,0008
24	10,6752	0,8394	0,8351	0,8329	0,8306	0,8327	0,8326	0,0008
26	12,2822	0,9102	0,9047	0,9062	0,9044	0,9055	0,9063	0,0009
28	13,6749	0,9710	0,9674	0,9668	0,9667	0,9682	0,9684	0,0008
30	15,5557	1,0603	1,0555	1,0555	1,0544	1,0528	1,0548	0,0009

Standard Deviation (δ2)								
Robots per task	Optimal	λ_B=1.0	λ_B=0.8	λ_B=0.6	λ_B=0.4	λ_B=0.2	λ_B=0.0	Greedy
2	0,0200	0,0070	0,0071	0,0070	0,0071	0,0071	0,0072	0,0001
4	0,2886	0,0660	0,0640	0,0621	0,0579	0,0583	0,0565	0,0002
6	0,3054	0,0789	0,0873	0,0898	0,0909	0,0906	0,0902	0,0003
8	0,4153	0,0999	0,1179	0,1187	0,1219	0,1235	0,1281	0,0004
10	0,4304	0,1120	0,1369	0,1476	0,1474	0,1443	0,1432	0,0004
12	0,7315	0,1911	0,1902	0,1840	0,1835	0,1814	0,1839	0,0004
14	0,7778	0,2184	0,2137	0,2039	0,1939	0,1925	0,1855	0,0003
16	0,9454	0,2301	0,2150	0,1958	0,1932	0,1848	0,1805	0,0004
18	0,5937	0,0560	0,0563	0,0552	0,0521	0,0592	0,0584	0,0003
20	0,7360	0,0672	0,0678	0,0684	0,0595	0,0598	0,0603	0,0003
22	0,8237	0,0746	0,0759	0,0696	0,0651	0,0675	0,0667	0,0002
24	0,9058	0,0987	0,0926	0,0836	0,0810	0,0861	0,0859	0,0003
26	1,0197	0,0857	0,0857	0,0858	0,0859	0,0853	0,0861	0,0003
28	1,2254	0,0909	0,0906	0,0909	0,0919	0,0924	0,0921	0,0003
30	1,2768	0,1481	0,1502	0,1526	0,1512	0,1503	0,1516	0,0003

Median								
Robots per task	Optimal	λ_B=1.0	λ_B=0.8	λ_B=0.6	λ_B=0.4	λ_B=0.2	λ_B=0.0	Greedy
2	0,1630	0,0299	0,0295	0,0295	0,0296	0,0295	0,0296	0,0002
4	0,6631	0,1067	0,1073	0,1065	0,1057	0,1054	0,1059	0,0004
6	1,8231	0,3649	0,3535	0,3421	0,3437	0,3342	0,3261	0,0006
8	2,8456	0,5104	0,4992	0,4893	0,4892	0,4625	0,4525	0,0007
10	3,8080	0,6536	0,6327	0,6205	0,5864	0,5694	0,6011	0,0008
12	4,5306	0,6517	0,5531	0,5021	0,4689	0,4526	0,4465	0,0008
14	5,3464	0,6184	0,5140	0,4987	0,4984	0,4984	0,4881	0,0007
16	6,2362	0,6120	0,5880	0,5811	0,5742	0,5660	0,5615	0,0008
18	6,6783	0,6230	0,6216	0,6211	0,6198	0,6214	0,6211	0,0007
20	7,9123	0,6983	0,6984	0,6980	0,6959	0,6957	0,6982	0,0008
22	9,0619	0,7461	0,7422	0,7403	0,7401	0,7410	0,7406	0,0007
24	10,6192	0,8208	0,8162	0,8179	0,8146	0,8162	0,8151	0,0008
26	12,2581	0,8980	0,8905	0,8903	0,8891	0,8901	0,8906	0,0008
28	13,8032	0,9651	0,9584	0,9582	0,9570	0,9569	0,9568	0,0008
30	15,5608	1,0338	1,0271	1,0259	1,0256	1,0241	1,0280	0,0008
