# Supplementary material for: Multi-Robot Coalitions Formation with Deadlines: Complexity Analysis and Solutions
Source: PLoS One. 2017 Jan 24;12(1):e0170659. doi: 10.1371/journal.pone.0170659 (PMC5261615; doi:10.1371/journal.pone.0170659)
Supplement: S5 Table — These results show the ratio between the utility obtained with MDRA and the utility of the optimal strategy. (PDF) [file pone.0170659.s005.pdf]

Mean, standard deviation and median of the execution with hard deadline.

Ratio between utility obtained with MDRA and the utility of the optimal strategy.

Mean							
Number of Robots	λ_B=1.0	λ_B=0.8	λ_B=0.6	λ_B=0.4	λ_B=0.2	λ_B=0.0	Greedy
12	0,4743	0,4984	0,4856	0,4872	0,4872	0,4159	0,4938
16	0,5599	0,5576	0,5404	0,5354	0,5354	0,4845	0,4995
20	0,5793	0,5913	0,5845	0,5829	0,5829	0,5512	0,4836
24	0,6203	0,6345	0,6224	0,6178	0,6178	0,5964	0,4937
28	0,6258	0,6477	0,6386	0,6354	0,6354	0,6322	0,4932
32	0,6398	0,6586	0,6583	0,6528	0,6528	0,6504	0,5128
36	0,6531	0,6760	0,6666	0,6616	0,6616	0,6600	0,5212
40	0,6648	0,6843	0,6797	0,6743	0,6743	0,6735	0,5394

Standard Deviation (δ2)							
Number of Robots	λ_B=1.0	λ_B=0.8	λ_B=0.6	λ_B=0.4	λ_B=0.2	λ_B=0.0	Greedy
12	0,1046	0,0999	0,0952	0,0956	0,0956	0,0968	0,0636
16	0,0826	0,0874	0,0875	0,0872	0,0872	0,0883	0,0467
20	0,0605	0,0624	0,0671	0,0649	0,0649	0,0652	0,0411
24	0,0524	0,0494	0,0543	0,0535	0,0535	0,0583	0,0412
28	0,0481	0,0491	0,0482	0,0470	0,0470	0,0466	0,0374
32	0,0435	0,0445	0,0444	0,0447	0,0447	0,0460	0,0354
36	0,0454	0,0465	0,0489	0,0478	0,0478	0,0478	0,0357
40	0,0449	0,0409	0,0422	0,0416	0,0416	0,0420	0,0377

Median							
Number of Robots	λ_B=1.0	λ_B=0.8	λ_B=0.6	λ_B=0.4	λ_B=0.2	λ_B=0.0	Greedy
12	0,5103	0,5093	0,4858	0,4871	0,4871	0,4410	0,4816
16	0,5569	0,5719	0,5474	0,5360	0,5360	0,4885	0,4865
20	0,5715	0,5947	0,6317	0,6108	0,6108	0,5637	0,4748
24	0,6046	0,6483	0,6483	0,6404	0,6404	0,6032	0,4823
28	0,5790	0,6998	0,6507	0,6459	0,6459	0,6372	0,4940
32	0,5975	0,7130	0,6963	0,6904	0,6904	0,6904	0,5078
36	0,6119	0,7378	0,7077	0,6933	0,6933	0,6923	0,5089
40	0,6234	0,7370	0,7217	0,7161	0,7161	0,7161	0,5214
