# Supplementary material for: Multi-Robot Coalitions Formation with Deadlines: Complexity Analysis and Solutions
Source: PLoS One. 2017 Jan 24;12(1):e0170659. doi: 10.1371/journal.pone.0170659 (PMC5261615; doi:10.1371/journal.pone.0170659)
Supplement: S6 Table — These results show the ratio between the utility obtained with MDRA and the utility of the optimal strategy. (PDF) [file pone.0170659.s006.pdf]

Mean, standard deviation and median of the execution with hard deadline using the 50 environments with the highest deadline values.

Ratio between utility obtained with MDRA and the utility of the optimal strategy.

Mean							
Number of Robots	λ_B=1.0	λ_B=0.8	λ_B=0.6	λ_B=0.4	λ_B=0.2	λ_B=0.0	Greedy
12	0,5284	0,5396	0,5068	0,4882	0,4882	0,4726	0,4685
16	0,6809	0,6525	0,6540	0,6491	0,6491	0,6254	0,4470
20	0,6587	0,6854	0,6859	0,6571	0,6571	0,6407	0,4738
24	0,6878	0,7012	0,6900	0,6640	0,6640	0,6485	0,5040
28	0,6875	0,7269	0,6764	0,6586	0,6586	0,6586	0,5166
32	0,7292	0,7544	0,7297	0,7237	0,7237	0,7237	0,5464
36	0,7348	0,7727	0,7624	0,7502	0,7502	0,7502	0,5598
40	0,7575	0,7885	0,7787	0,7621	0,7621	0,7621	0,5795

Standard Deviation (δ2)							
Number of Robots	λ_B=1.0	λ_B=0.8	λ_B=0.6	λ_B=0.4	λ_B=0.2	λ_B=0.0	Greedy
12	0,0763	0,0751	0,0673	0,0784	0,0784	0,0776	0,0447
16	0,0607	0,0671	0,0649	0,0666	0,0666	0,0567	0,0605
20	0,0443	0,0341	0,0440	0,0479	0,0479	0,0472	0,0532
24	0,0439	0,0339	0,0474	0,0501	0,0501	0,0490	0,0492
28	0,0494	0,0345	0,0453	0,0452	0,0452	0,0452	0,0540
32	0,0401	0,0424	0,0417	0,0417	0,0417	0,0417	0,0510
36	0,0287	0,0300	0,0384	0,0400	0,0400	0,0400	0,0452
40	0,0351	0,0320	0,0367	0,0376	0,0376	0,0376	0,0500

Median							
Number of Robots	λ_B=1.0	λ_B=0.8	λ_B=0.6	λ_B=0.4	λ_B=0.2	λ_B=0.0	Greedy
12	0,5702	0,5702	0,4654	0,4456	0,4456	0,4229	0,3860
16	0,7522	0,7361	0,7080	0,7080	0,7080	0,6851	0,3338
20	0,7155	0,7539	0,7307	0,7089	0,7089	0,6982	0,4780
24	0,7740	0,7632	0,7443	0,7133	0,7133	0,6998	0,4960
28	0,7830	0,7816	0,7028	0,6482	0,6482	0,6482	0,4837
32	0,7844	0,7888	0,7482	0,7053	0,7053	0,7053	0,5125
36	0,7820	0,7857	0,7753	0,7564	0,7564	0,7564	0,5386
40	0,7848	0,7994	0,7857	0,7740	0,7740	0,7740	0,5501
